# Galloyl-RGD as a new cosmetic ingredient

**DOI:** 10.1186/1471-2091-15-18

**Published:** 2014-08-08

**Authors:** Dae-Hun Park, Dae Hyun Jung, Soo Jung Kim, Sung Han Kim, Kyung Mok Park

**Affiliations:** 1 Department of Oriental Medicine Materials, Dongshin University, Naju, Jeonnam 520-741, Korea; 2 BIO-FD&C Co., Ltd., JBRC (BBI), 121, Naepyung, Hwasun, Jeonnam 519-801, Korea; 3 Nutrex Technology Co., Ltd., Carden 5 Tool BF S-21, 292 Munjeong, Songpa, Seoul 138-962, Korea; 4 Department of Pharmaceutical Engineering, Dongshin University, Naju, Jeonnam 520-741, Korea

## Abstract

**Background:**

The cosmetics market has rapidly increased over the last years. For example, in 2011 it reached 242.8 billion US dollars, which was a 3.9% increase compared to 2010. There have been many recent trials aimed at finding the functional ingredients for new cosmetics. Gallic acid is a phytochemical derived from various herbs, and has anti-fungal, anti-viral, and antioxidant properties. Although phytochemicals are useful as cosmetic ingredients, they have a number of drawbacks, such as thermal stability, residence time in the skin, and permeability through the dermal layer. To overcome these problems, we considered conjugation of gallic acid with a peptide.

**Results:**

We synthesized galloyl-RGD, which represents a conjugate of gallic acid and the peptide RGD, purified it by HPLC and characterized by MALDI-TOF with the aim of using it as a new cosmetic ingredient. Thermal stability of galloyl-RGD was tested at alternating temperatures (consecutive 4°C, 20°C, or 40°C for 8 h each) on days 2, 21, 41, and 61. Galloyl-RGD was relatively safe to HaCaT keratinocytes, as their viability after 48 h incubation with 500 ppm galloyl-RGD was 93.53%. In the group treated with 50 ppm galloyl-RGD, 85.0% of free radicals were removed, whereas 1000 ppm galloyl-RGD suppressed not only L-DOPA formation (43.8%) but also L-DOPA oxidation (54.4%).

**Conclusions:**

Galloyl-RGD is a promising candidate for a cosmetic ingredient.

## Background

The worldwide cosmetics market reached 242.8 billion US dollars in 2011, which was a 3.9% increase compared to 2010 [1]. There are many compounds used as cosmetic ingredients, such as phycobiliprotein from natural sources, which is used as a colorant [2], polysaccharides used as emulsifiers [3], and a polymer used for mascara [4].

Much research has been conducted to develop appropriate materials for cosmetic ingredients. In particular, these studies focused not only on beauty care, but also on functional aspects. Many trials are underway that aim to find ingredients for functional cosmetics, which would have whitening [5], anti-oxidant [6] or anti-ageing [7] effects. Any cosmetic ingredient has to satisfy several requirements, such as thermal stability, high dermal absorption rate, and perfume.

Gallic acid (3,4,5-trihydroxybenzoic acid) is a phenolic acid and a phytochemical derived from herbs. It is found in gallnuts, sumac, witch hazel, tea leaves, oak bark, and other plants [8], and has anti-fungal [9], anti-viral [10], and antioxidant [11] properties, which are useful for a cosmetic ingredient.

With the development of biotechnology, peptides can be now produced on a large scale massively produced, and are used in cosmetic industry as ingredients. Peptides used in topical anti-ageing products are classified into 4 categories: carrier peptides, signaling peptides, enzyme inhibitors, and neurotransmitter inhibitors [12]. Carrier peptides can deliver other components of cosmetic preparations when these are applied topically.

In this study, we explored the possibility of synthesis of a phytochemical (gallic acid) and a peptide for use as a cosmetic ingredient. We assessed 3 aspects of the novel compound (galloyl-RGD): its safety, stability, functionality as a cosmetic ingredient. To evaluate the safety of galloyl-RGD to the skin, we measured the viability of HaCaT keratinocytes. To assess its stability, we measured its thermal stability. We also analysed its free radical–scavenging effect, and its ability to inhibit L-DOPA formation and L-DOPA oxidation.

## Results

### Galloyl-RGD purification using a C18 preparative column

**Figure 1:**
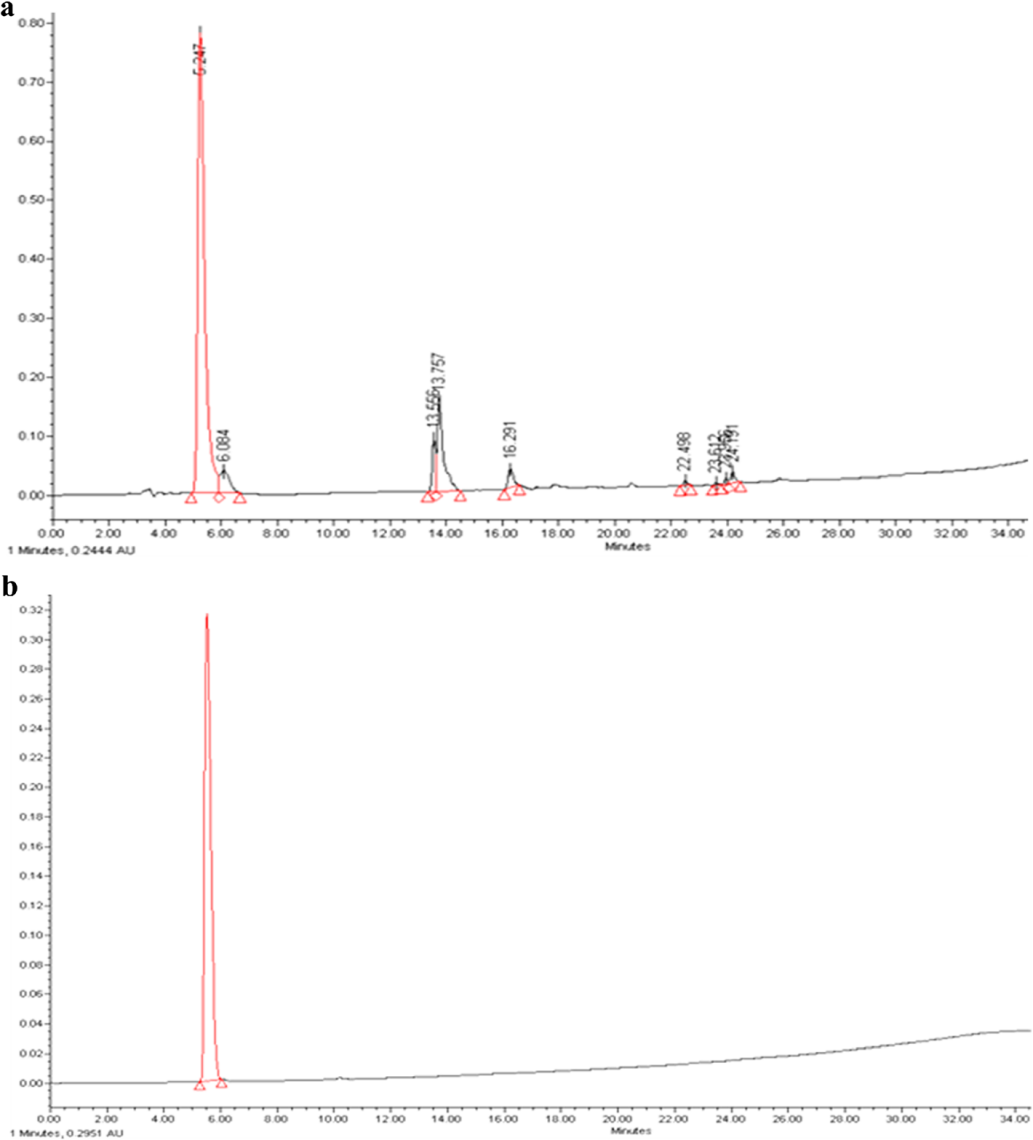
**Galloyl-RGD purification by HPLC. (a)** Before HPLC purification using a C18 preparative column there were many compounds which the rate in galloyl-RGD was about 30% and were shown 8 peaks at least. **(b)** Purity of galloyl-RGD increased to 95% after HPLC purification using a C18 preparative column and was detected only 1 peak.

We developed a method for synthetic peptide purification by high performance liquid chromatography (HPLC) on an analytical C18 column (250 × 4.60 mm, 5 μm; detection wavelength, 230 nm; flow rate, 1 mL/min) in a gradient using 0.1% trifluoroacetic acid in water and 0.1% trifluoroacetic acid in acetonitrile as solvents. Although the purity of synthetic galloyl-RGD was about 70% (Figure 1a), it increased up to 95% after HPLC purification (Figure 1b).

### Confirmation of galloyl-RGD structure by MALDI-TOF

**Figure 2:**
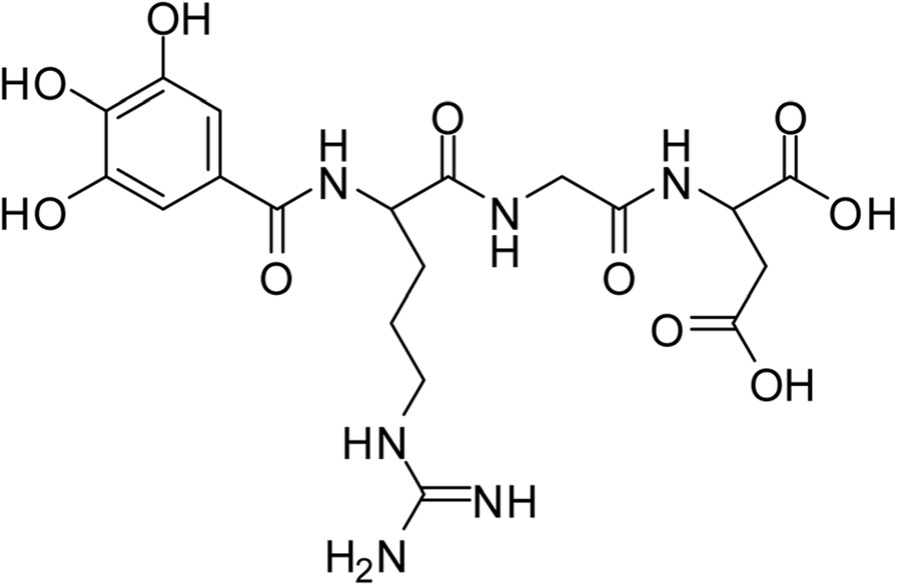
**The chemical structure of galloyl-RGD.** The chemical formula of galloyl-RGD is C_19_H_26_N_6_O_10_ and the molecular weight of it is 498.44.

**Figure 3:**
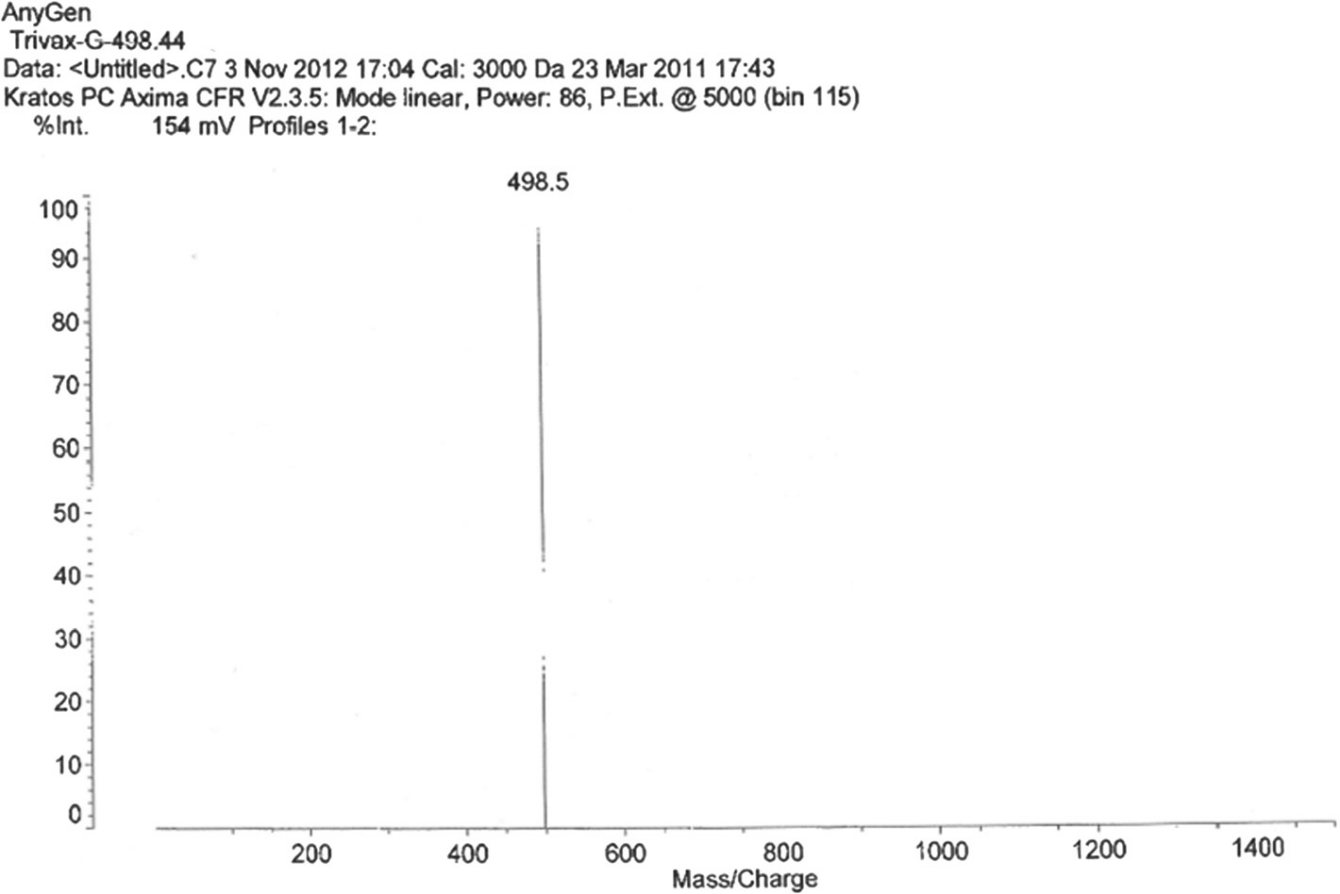
**Galloyl-RGD identification using MALDI-TOF mass spectrometry.** Galloyl-RGD was purified by HPLC (95% pure), and its molecular weight and chemical structure were identified by MALDI-TOF.

Galloyl-RGD was synthesized to combine RGD as a peptide to support physiological activity and gallic acid as a phytochemical to scavenge free radicals. A MALDI-TOF mass spectrometry assay (linear mode, α-cyano-4-hydroxy-cinnamic acid matrix) was conducted to confirm the molecular weight and chemical structure of galloyl-RGD (Figure 2). As shown in Figure 3, the galloyl-RGD produced met the quality standards required for a cosmetic material.

### Galloyl-RGD is stable for 60 days at alternating temperatures

Storage conditions of cosmetics may change from low temperature (in a refrigerator) to room temperature (although most of them are kept at room temperature), i.e. they may be subjected to temperature changes of ca. 20°C. For this reason, thermal stability is an important characteristic of cosmetic materials.

Both gallic acid and galloyl-RGD were stable at alternating temperatures, but their stabilities were different depending on the storage period. The amounts remaining after storage for 20 days, 40 days, or 60 days were 81 ± 4%, 74 ± 6%, and 66 ± 23%, respectively, for gallic acid, and 94 ± 4%, 92 ± 8%, and 87 ± 29% for galloyl-RGD (Table 1).

**Table 1:** Stability of galloyl-RGD during 60-day storage

**Storage duration**	**Gallic acid**	**Galloyl-RGD**
1 Day	99 ± 2%,	99 ± 2%,
20 Days	81 ± 4%,	94 ± 4%,
40 Days	74 ± 6%,	92 ± 8%,
60 Days	66 ± 2%	87 ± 2%

Gallic acid was relatively unstable, and was degraded to 66 ± 2% on the end of the experiment, whereas galloyl-RGD was degraded by only 87 ± 2%.

### Galloyl-RGD is safe to HaCaT keratinocytes

**Figure 4:**
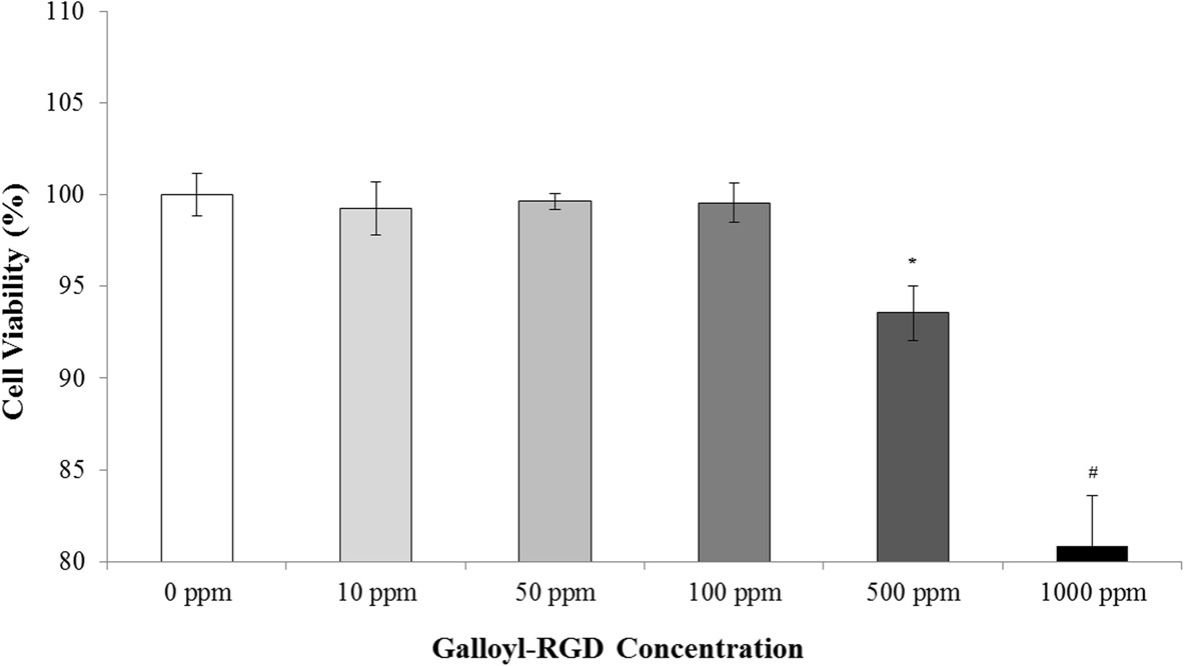
**Safety of galloyl-RGD for HaCaT keratinocytes.** After treatment with 100 ppm galloyl-RGD for 48 h, HaCaT keratinocyte viability was 99.56%. * p < 0.01 vs. 0 ppm group to 100 ppm group; # *p* < 0.01 vs. all groups.

Human body is surrounded by keratinocytes, which protect it from various factors and prevent damage from chemicals, microbes and dehydration [13]. Thus, development of new cosmetics should include measurements of their effects on keratinocytes.After treatment for 48 h, the viability of HaCaT keratinocytes was 99.24%, 99.65%, 99.56%, 93.53%, and 80% in the presence of 10 ppm, 50 ppm, 100 ppm, 500 ppm, and 1000 ppm galloyl-RGD, respectively (Figure 4). These results indicate that galloyl-RGD is safe to HaCaT keratinocytes at concentrations of up to 100 ppm.

### Galloyl-RGD scavenges free radicals produced by DPPH and reactive oxygen species by UV irradiation

**Figure 5:**
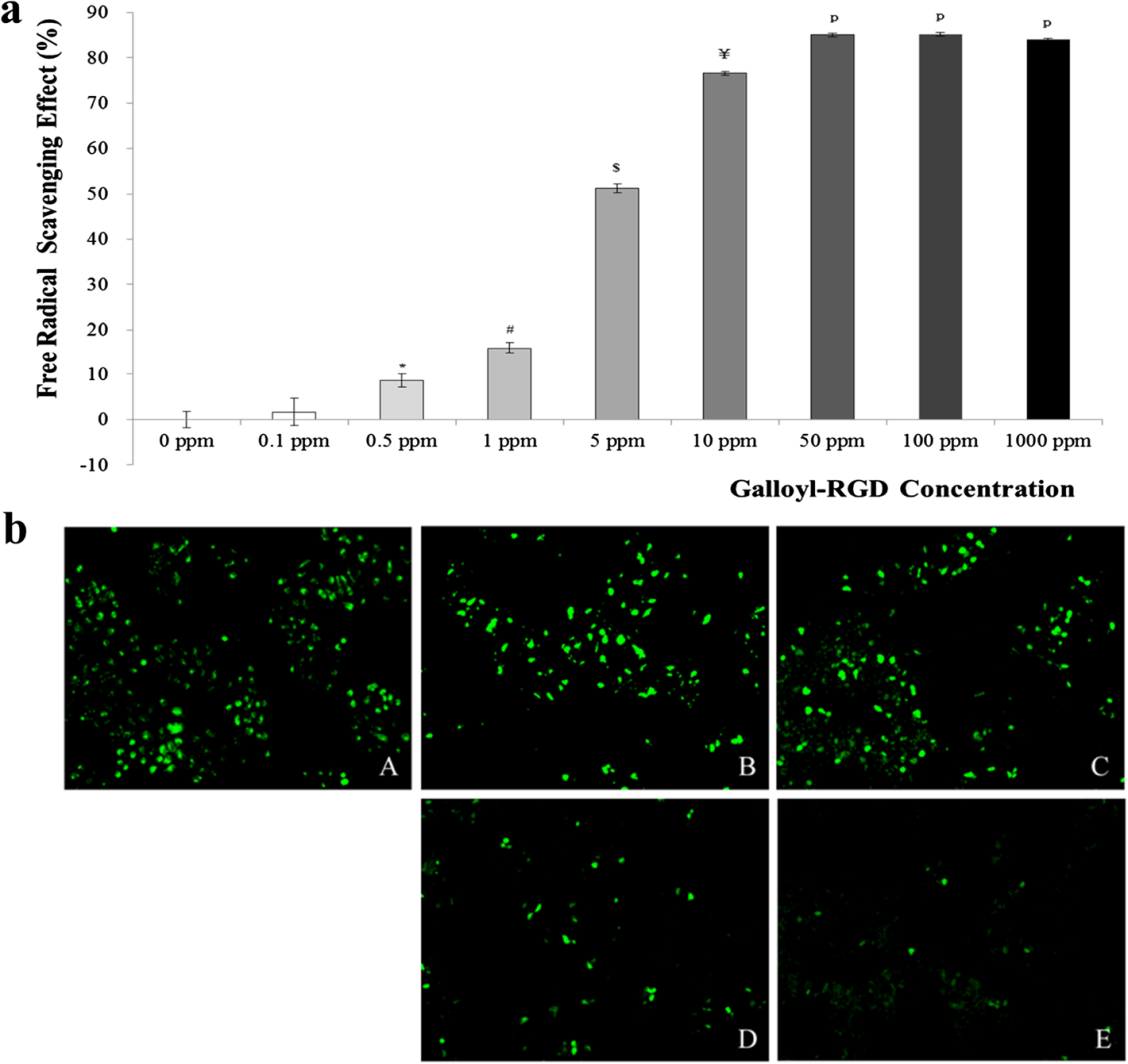
**Galloyl-RGD effectively scavenged DPPH both in test tube and ROS in HaCaT keratinocytes. (a)** Galloyl-RGD is an efficient DPPH scavenger. After 48 h in 10 ppm galloyl-RGD, DPPH was decreased to 76.6%; galloyl-RGD scavenged DPPH in a dose-dependent manner. **(b)** Galloyl-RGD efficiently eliminated ROS generated by ultraviolet radiation in HaCaT keratinocytes. The ROS was scavenged more effectively by 50 ppm galloyl-RGD treatment or more than by 20 ppm NAC treatment. (A) 20 ppm N-acetyl-L-crystein treated group; (B) 0 ppm galloyl-RGD treated group; (C) 25 ppm galloyl-RGD treated group; (D) 50 ppm galloyl-RGD treated group; (E) 100 ppm galloyl-RGD treated group.

Free radicals have both useful and harmful effects on organisms; they confer protection to host against harmful fungi, bacteria, and viruses, but may also induce host cell damage [14]. In the case of cosmetics, damage to epithelial cells in the skin is more important than other effects because most cosmetics are directly applied to the skin. The levels of free radicals are controlled by the balance of their production and elimination in the cells. A reduction in elimination relative to production could destroy epithelial cells of the skin.We found that the free radical (DPPH)–scavenging effect of galloyl-RGD after 48 h incubation was 76.6% at 10 ppm, and the quantities of free radicals decreased with increasing galloyl-RGD concentrations in a dose-dependent manner (Figure 5a). Galloyl-RGD scavenged effectively free radical not only in the test tube but also efficiently eliminated ROS which was generated by 60 min ultraviolet irradiation in HaCaT cells (Figure 5b). The ROS scavenging effect of galloyl-RGD both in 50 ppm and 100 ppm galloyl-RGD treatment groups was higher than in 20 ppm NAC treatment.

### Galloyl-RGD efficiently inhibits L-DOPA formation and L-DOPA oxidation

**Figure 6:**
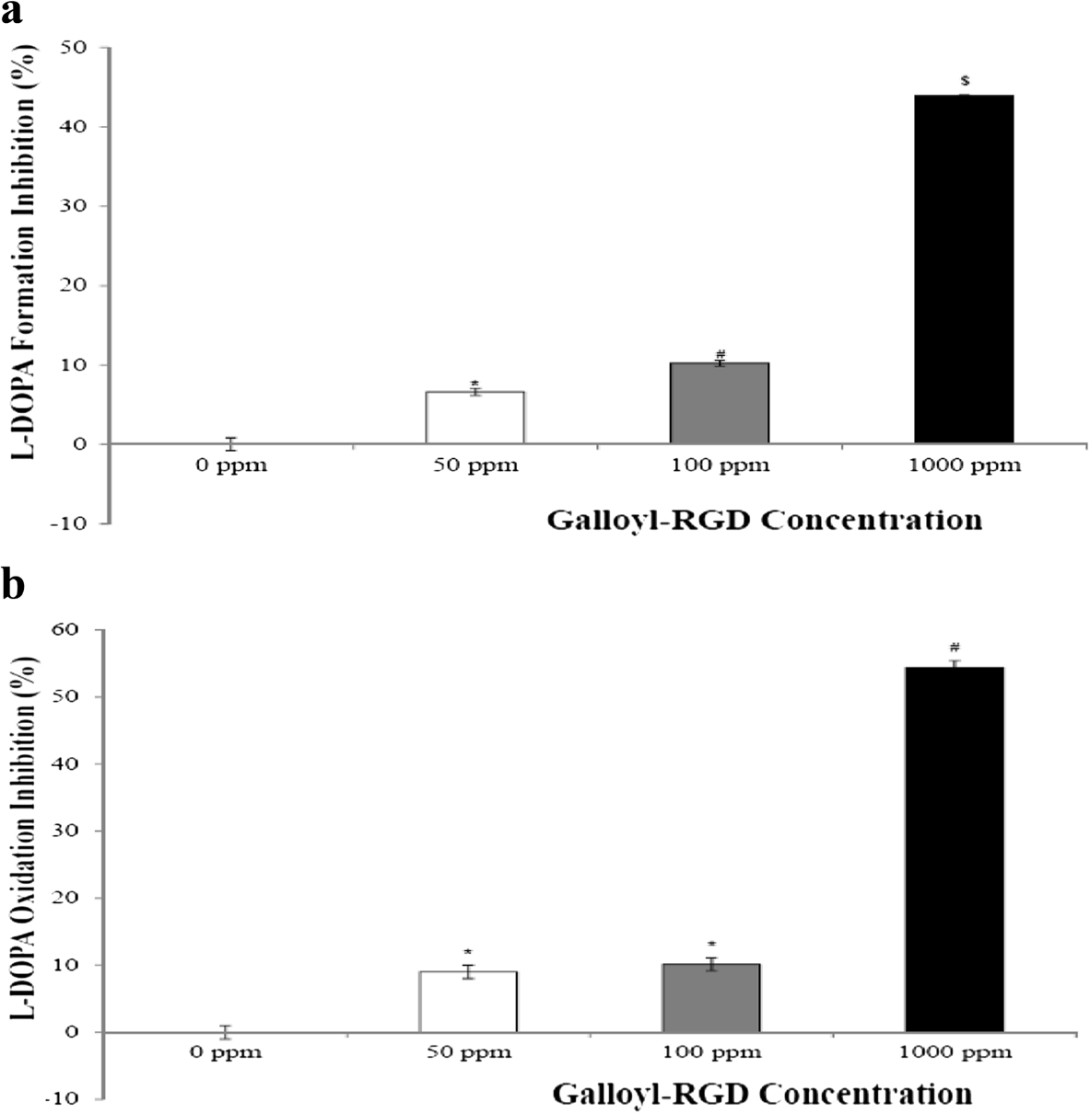
**Galloyl-RGD effectively inhibits tyrosinase, thus retarding the appearance of intermediates of melanin synthesis. (a)** Tyrosine conversion to L-DOPA. After 48 h in the presence of 1000 ppm galloyl-RGD, DOPA appearance in the treated group was inhibited to 43.8%. **(b)** DOPA conversion to dopaquinone (an oxidized form of L-DOPA and a melanin precursor). After 48 h in the presence of 1000 ppm galloyl-RGD, L-DOPA oxidation in the treated group was decreased to 54.4%. * p < 0.01 vs. 0 ppm group; # *p* < 0.01 vs. 0 ppm group to 50 ppm group; $ *p* < 0.01 vs. all groups.

Tyrosinase converts tyrosine to 3,4-dihydroxyphenylalanine (L-DOPA), and if L-DOPA is oxidized it is converted into dopaquinone, which is a precursor of melanin biosynthesis [15]. Melanin is a pigment related to skin darkening. Higher amounts of the above compounds result in larger black spots on the skin. The effect on melanin synthesis is one of the important parameters that need to be tested during development of new cosmetics.We measured L-DOPA formation inhibition and suppression of L-DOPA oxidation after incubation with galloyl-RGD for 48 h. The L-DOPA formation inhibition rate was 6.6%, 10.2%, and 43.8% in the presence of 50 ppm, 100 ppm and 1000 ppm galloyl-RGD, respectively (Figure 6a). As shown in Figure 6b, galloyl-RGD suppressed L-DOPA oxidation to 9.0%, 10.1%, and 54.4% at 50 ppm, 100 ppm or 1000 ppm concentrations, respectively, compared to the control group.

## Discussion

Galloyl-RGD is a synthetic conjugate of gallic acid and a peptide, synthesized for intended use as a new cosmetic ingredient, purified using HPLC and validated by MALDI-TOF mass spectrometry. Thermal stability of galloyl-RGD was tested at alternating temperatures (consecutive 4°C, 20°C and 40°C [8 h each] on days 2, 21, 41, and 61). It was relatively safe to HaCaT keratinocytes, as their viability after 48 h incubation was 93.53% in the presence of 500 ppm galloyl-RGD. In the 50 ppm galloyl-RGD–treated group, 85.0% of free radical was removed, ROS was scavenged more effectively more than by 20 ppm NAC-treated group in HaCaT cells, and treatment with 1000 ppm galloyl-RGD suppressed not only activation-DOPA formation (43.8%) but also L-DOPA oxidation (54.4%).

To pursue the beauty is a basic human instinct, and as people were looking for ways to make themselves more beautiful the cosmetic industry has significantly grown. Recently, the number of trials aimed at finding more effective ingredients for cosmetics has increased, especially search for the functional ingredients effective for whitening [5], smoothing the creases [16], scavenging free radicals [6], or anti-ageing [7].

For a long time, natural products have been used for multiple purposes such as remedies, cosmetics, culinary materials, or additives; these various functions are due to multiple phytochemicals in the natural products. A number of recent studies have found that the functions of natural products depend on constituent phytochemicals [17-22].

Gallic acid is a phytochemical, which has been isolated from several plants such as *Emblica officinalis* Gaertn [23], *Phaleria macrocarpa* Boerl [24], *Quercus robur *[25], and *Castanea sativa* L. [26] and has anti-fungal [9], anti-viral [10], antioxidant [11] properties. However, gallic acid is thermally unstable [27], which makes its use as cosmetic ingredient difficult.

To overcome this weakness, we considered gallic acid conjugation with a peptide. Recently, many peptides have been introduced, as they not only have important functions in specific fields, but are also able to improve something more effective. For example, peptides have been used in cosmetic industry to increase skin permeability and duration [12].

## Conclusions

We conclude that galloyl-RGD is a promising candidate for a cosmetic ingredient.

## Methods

### Galloyl-RGD synthesis and purification

Galloyl-RGD was synthesized with 9-fluorenylmethoxycarbonyl (as an amino acid protector against aminolysis) by solid-phase peptide synthesis, linked with amino acid residues using N-hydroxybenzotriazol-N,N-di-cyclohexylcarbodiimide, and then purified by reverse-phase HPLC (Waters, MA, USA; column: Gemini C18 110 Å 250 × 21.2 mm) (Figure 1a & b) in a gradient of acetonitrile in 0.1% trifluoroacetic acid. A MALDI-TOF mass spectrometry assay (linear mode, α-cyano-4-hydroxy-cinnamic acid matrix) was performed to ensure the synthetic quality of galloyl-RGD (molecular weight and chemical structure). Briefly, Axima CFR™ (Kratos Analytical Ltd., Japan) was used to conduct MALDI-TOF assay with 8.0 × 10^-4^ Pascal Gauge Pressure, Linear mode, and 96 square well sample plate. The purified galloyl-RGD was combined with an equal volume of alpha-cyano-4-hydroxycinnamic acid solution (10 mg/ml CHCA in 50:50 water/Acetonitrile solution) and spotted onto a MALDI target. MALDI-MS data was acquired using Axima-CFR™ Plus-mass spectrometer in positive ion reflectron mode.

### Stability at alternating temperatures

Galloyl-RGD was exposed consecutively to 4°C, 20°C, and 40°C (8 h each) on days 2, 21, 41, and 61, and quantified by HPLC.

### Cell safety assessment

HaCaT keratinocytes were seeded in 24-well plates (5 × 10^3^ cells/well) in triplicate and treated with 10, 50, 100, or 1000 ppm galloyl-RGD for 48 h; control cells were treated with DMEM with 10% FBS. Cell proliferation was then analysed later using the MTT method.

### Analysis of the free radical–scavenging effect

Galloyl-RGD (0–1000 ppm) was mixed with 0.1 mM 1,1-diphenyl-2-picryl hydrazyl (DPPH; Sigma-Aldrich, St. Louis, MO, USA.) in ethanol, stirred vigorously with a vortex mixer for 10 s, and then incubated for 30 min in a refrigerator. The results of DPPH scavenging were determined using an ELISA reader (Biochrom Ltd., Cambridge, UK). In order to analyze ROS scavenging effect of galloyl-RGD, HaCaT keratinocytes were used and intracellular ROS levels were investigated using with the oxidation-sensitive fluorescent probe, 2',7'-dichlorofluorescein diacetate (DCF-DA, Sigma-Aldrich). Cells (1 × 10^4^ cells/well) were cultured in 24-well plate for 12 h and then treated with 20 ppm N-Acetyl-L-cysteine (NAC, Sigma-Aldrich) or with various concentrations of galloyl-RGD (0, 25, 50, and 100 ppm) for 24 h. In order to generate intracellular ROS in cells they were exposed to 40 mJ/cm^2^ ultraviolet radiations for 60 min using with ultraviolet radiation (Crosslinker Model BLX-254, VILBER Lourmat, France). After irradiation they were washed with PBS and incubated with 10 μM 2′,7′-Dichlorofluorescin diacetate (Sigma-Aldrich) for 30 min at 37°C. The cellular fluorescent images were obtained using with Axiovert 40 cfl (Carl Zeiss, Göttingen, Germany).

### L-DOPA formation analysis

The inhibitory effect of galloyl-RGD on tyrosinase activity was analysed using mushroom tyrosinase according to Vallisuta *et al*. [28]. The sample (20 μL), 0.1 M phosphate buffered saline (220 μL) and tyrosinase (20 μL; 1500–2000 U/mL) were mixed, 1.5 mM tyrosine solution (40 μL) was added, and the mixture was incubated for 15 min at 37°C. Tyrosinase activity was measured as absorbance at 492 nm using an ELISA reader. Phosphate buffered saline was used as a substrate and control.

The percentage of tyrosinase inhibition was calculated using the following equation:

Tyrosinase Inhibition Activity (%) = [1 – A/B] × 100, where ‘A’ is the difference between the optical density (OD) value of the sample and that of the substrate, and ‘B’ is the difference between the OD value of the mixture of mushroom tyrosinase and the substrate and that of the substrate alone.

### Analysis of L-DOPA oxidation inhibition

Inhibition of L-DOPA oxidation by galloyl-RGD was assessed based on a modification of the tyrosinase inhibition assay. The sample (50 μL), 0.1 M phosphate buffered saline (850 μL) and mushroom tyrosinase (50 μL; 1500–2000 U/mL) were mixed, 1.5 mM tyrosine solution (40 μL) was added, and the mixture was incubated for 6 min at 37°C. L-DOPA (50 μL; 0.06 mM) was then added, and the mixture was incubated for 1 min at 37°C and measured using ELISA (wavelength: 475 nm). Phosphate buffer saline was used as a substrate and control.

The percentage of L-DOPA oxidation inhibition effect was calculated using the following equation:

L-DOPA Oxidation Inhibition Effect (%) = [1 – A/B] × 100, where ‘A’ is the difference between the OD value of the sample and that of the substrate, and ‘B’ is the difference between the OD value of the mixture without L-DOPA and that of the substrate.

### Statistical analysis

Differences between groups were evaluated by one-way analysis of variance followed by Dunnett’s multiple comparison test; *p* < 0.01 was considered significant.

## Competing interests

The authors declare that they have no competing interests.

## Authors’ contributions

DHP drafted the manuscript, participated in the performed the statistical analysis, and the interpreted the data. DHJ carried out the galloylgalloyl-RGD analysis using with HPLC and SJK participated in the confirmed the galloylgalloyl-RGD and the characterized the temperature stability. SHK carried out the experiments such as cell viability measurement, analyzing the free radical scavenging effect, and inhibiting effect against L-DOPA formation and L-DOPA oxidation. KMP conceived of the study, and participated in its design and coordination and helped to draft the manuscript. All authors read and approved the final manuscript.
